# From laboratory targets to experiential authenticity: reframing the study of veridicality in OBEs

**DOI:** 10.3389/fpsyg.2026.1720998

**Published:** 2026-06-02

**Authors:** Marina Weiler, Marieta Pehlivanova, Philip J. Cozzolino

**Affiliations:** Department of Psychiatry and Neurobehavioral Sciences, University of Virginia Health System, Charlottesville, VA, United States

**Keywords:** altered states of consciousness, experimental methodology, extra-sensory perception, non-local consciousness, veridical perception

## Abstract

Out-of-body experiences (OBEs) are altered states sometimes accompanied by reports of veridical perception—accurate information seemingly obtained from a vantage point outside the body and ostensibly without conventional sensory input. While case collections and early laboratory studies suggest this possibility, replication under conventional laboratory protocols has been challenging. This article reviews the evidentiary landscape and argues that standard target-identification paradigms—often conducted in sterile settings with inexperienced participants—misalign with the dynamics of the OBE state. We identify five key determinants that constrain laboratory outcomes: (1) the participant’s mental/emotional state, (2) the participant–researcher relationship, (3) researcher intention, (4) variability in participant skill, and (5) the intrinsic purpose of the experience. We propose reframing methodology around these determinants, complementing lab tasks with rigorously vetted spontaneous cases. Together, these adjustments can increase ecological validity while preserving falsifiability, offering a pathway toward more effective investigation of veridical perception in OBEs and the implications for models of consciousness, using structured case evaluation and independent verification criteria.

## Introduction

Out-of-body experiences (OBEs) are altered states of consciousness in which individuals report a sense of separation from their physical bodies, often accompanied by vivid sensory perceptions and experiences of the environment that are consistent with this external viewpoint (Alvarado, 2000; Weiler et al., 2025b). These experiences are frequently described as emotionally impactful and psychologically transformative, sometimes contributing to long-lasting changes in beliefs, identity, or worldview (Shaw et al., 2026; Weiler et al., 2024). Among the most intriguing aspects of OBEs are claims of veridical perception—reports in which individuals appear to acquire accurate information about people, objects, or events from a vantage point outside the body. These perceptions apparently occur without conventional sensory input, as if through extra-sensory perception (ESP), the acquisition of information by means other than the five recognized sensory modalities—vision, hearing, touch, smell, and taste. Such accounts are particularly interesting to researchers exploring the mind-brain relationship, as they may challenge dominant materialist models of consciousness and support frameworks that allow for non-local cognition (Wahbeh et al., 2022; Weiler and Acunzo, 2025). A growing number of anecdotal reports outside laboratory conditions document OBEs in which individuals describe verified, specific details unknown to them at the time. Despite this, efforts to empirically validate these claims under controlled laboratory conditions have produced mixed results (see below for examples), and robust replication has proven difficult. This paper critically examines the limitations of traditional experimental approaches used to explore the possibility of ESP during OBEs. It outlines emerging methodological innovations aimed at more effectively investigating the veridical aspects of these experiences.

## OBEs as products of brain and cognitive processes

A number of researchers have proposed neurobiological and psychological explanations that interpret OBEs as internally generated perceptual phenomena rather than evidence of perception beyond the body. From this perspective, OBEs are understood as hallucinations or misperceptions produced by neural processes associated with disturbances in bodily self-representation (Woodhouse, 1994). Several neurological models suggest that extreme physiological stress, psychoactive agents, or near-fatal events may trigger OBEs through disruptions in temporal lobe activity or related neurochemical processes (Morse et al., 1989). Other influential frameworks emphasize the role of multisensory integration and vestibular processing, proposing that OBEs arise when the brain’s integration of visual, proprioceptive, and vestibular signals becomes disrupted, particularly within the temporoparietal junction (Blanke et al., 2005). Additional cognitive models attribute the vivid visual content of OBEs to processes of cognitive dedifferentiation, in which the brain constructs perceptual imagery from non-visual sensory information during altered states of consciousness (Terhune, 2009). Psychological theories have similarly emphasized alterations in body representation and dissociative processes. For example, previous studies proposed that reductions in proprioceptive input may destabilize the body schema and trigger experiences of disembodiment (Palmer, 1978a) or that OBEs arise when the brain constructs alternative “models of reality” based on memory and imagination when normal sensory input is disrupted (Blackmore, 1984).

Other researchers have suggested that OBEs may be associated with somatoform dissociation, absorption, or personality traits such as fantasy proneness and cognitive-perceptual schizotypy (Gow et al., 2004; Irwin, 1981, 2000). Related work has also reported that individuals who experience OBEs may show higher levels of body dissatisfaction, self-consciousness, and dissociative tendencies, potentially reflecting a more fragile or flexible sense of embodiment (Murray and Fox, 2005; Terhune, 2006). Together, these neurobiological and psychological models frame OBEs as phenomena arising from alterations in brain function, multisensory integration, or cognitive representations of the body, and they remain influential explanatory frameworks within mainstream neuroscience and psychology. However, one of the main challenges for neuropsychological explanations of OBEs is reports of verifiable perceptions of events or scenes that would have been inaccessible through normal sensory means, given the person’s physical condition or location.

## Empirical studies on the veridicality of OBEs

Since the early 20th century, researchers have attempted to empirically investigate the veridicality of OBEs—that is, the extent to which individuals reporting an OBE could perceive objective information from a spatial location outside their physical body. Although results have been mixed and often constrained by methodological limitations, these early investigations laid the foundation for contemporary approaches to the empirical study of OBEs.

### Hornell Hart’s early case studies

The earliest efforts to document veridical perception during OBEs relied on spontaneous case reports. Hart’s (1954) study represents an early and ambitious attempt to combine qualitative case analyses with quantitative methods, making it one of the first attempts to evaluate the veridicality of spontaneous OBEs systematically. Hart’s central hypothesis was that during OBEs, individuals might project a non-physical aspect of themselves—an “ESP body”—capable of perceiving distant or hidden information beyond the reach of normal sensory channels.

Analyzing over 100 spontaneous reports, Hart (1954) developed a veridicality index that rates each case according to its informational specificity, the availability of independent verification, and the degree to which ordinary explanatory mechanisms can be ruled out. These include all plausible non-ESP sources of information, such as sensory cues (visual, auditory, or tactile), prior knowledge, chance coincidence, memory error, inference, or unintentional information leakage. About one-third scored high, including striking instances of remote perception such as accurately describing the moment or circumstances of another person’s death from a distant location, later confirmed through independent verification. Although Hart acknowledged the limitations of spontaneous case reports—such as retrospective bias and the absence of controlled conditions—he used them as a foundation to argue for the plausibility of ESP projection as a real phenomenon.

### The Charles Tart studies with “Miss Z” and Robert Monroe

Moving beyond anecdotal case collections, Charles Tart was among the first researchers to systematically investigate the possibility of veridical perception during OBEs in controlled laboratory conditions. In his best-known early experiment (Tart, 1968), Tart studied, during four nights in a sleep laboratory, a young woman known as “Miss Z.” This case involved a young woman who first reported lifelong spontaneous OBEs while informally conversing with him outside the laboratory. She had never read about OBEs and believed the experiences were a normal part of sleep until learning otherwise as a teenager. After successfully conducting informal at-home trials in which she reportedly identified random numbers drawn before sleep, she agreed to spend four nights in Tart’s sleep laboratory, where her EEG, eye movements, skin resistance, and, on some nights, cardiovascular activity were continuously monitored. Each night, Tart generated a new five-digit random number using a table of random numbers and placed it face-up on a high shelf above the bed, where it was evident only from a position approximately 6.5 feet above the floor. Miss Z was instructed to try to have an OBE, and if she found herself “floating” near the ceiling, to look at the number, memorize it, and awaken to report it. No induction techniques were used. Over the four nights, she reported three incidents of “floating” and two instances of feeling completely out of her body. On the final night, she awakened at 6:04 am and correctly reported the target number (25132). Tart concluded that this incident provided suggestive evidence that the OBEs had parapsychological concomitants.

In another study, Tart (1998) studied Robert Monroe, another individual experienced in inducing OBEs, who participated in eight laboratory sessions. As in the “Miss Z” experiment, a five-digit number was placed on a high shelf in the sleep laboratory. Monroe did not correctly identify the target number; however, during one session, he provided a detailed description of the technician interacting with a man during the night. This interaction—unknown to any of the researchers at the time he reported it, as it was policy that no outsiders were allowed to see subjects or patients—occurred in a different room from the control console room, where the technician was supposed to be, while Monroe was connected to the physiological recording apparatus. The account was later verified: the technician’s husband had, in fact, made an unscheduled visit. Although the designated target was missed, the incidental accuracy of this observation led Tart to suggest that fixed, passive targets may not align with the attentional dynamics of the OBE state.

In his 1998 review (Tart, 1998), Tart presented four additional experiments conducted with different participants. Each participant was tasked with perceiving concealed visual targets—typically numbers, letters, or symbols—placed in distant or obscured locations. Across these studies, none of the participants successfully identified the target materials. Tart’s findings suggest that veridical perception, if it occurs during OBEs, may be selective and influenced by emotional salience or spontaneous attentional shifts rather than compliance with experimental instructions. The tendency of experiencers to report accurate but incidental environmental information points to the need for more ecologically valid designs and a broader conceptualization of what counts as veridical perception.

### The Osis and McCormick experiments with Alex Tanous (1970s–1980s)

Like Charles Tart, Karlis Osis conducted laboratory studies designed to test whether perceptual accuracy during OBEs reflected localized “externalized” sensing or conventional ESP (Osis, 1974). The participant, Dr. Alex Tanous, was not recruited for an experiment initially; he was selected after earlier spontaneous reports suggested he could induce OBEs voluntarily. In the laboratory tests, he lay in a distant soundproof room and was instructed to attempt an OBE and “position himself” at the viewing window of two custom optical devices that displayed randomly determined composite targets. Each target contained both (a) elements accessible through ESP and (b) an optical illusion component visible only from a single physical vantage point—directly in front of the device—thereby serving as the critical measure of localized perception. Tanous provided confidence ratings after each trial based on introspective cues he believed accompanied successful OBEs (e.g., feeling like a “point of light” or experiencing unity). Across both devices, overall scores and low-confidence trials were not significant, but high-confidence trials in the latter half of each study approached or reached statistical significance, including target aspects requiring localized viewing (*p* ≈ 0.05 in the Optical Device; *p* < 0.008 in the Color Wheel). These results were interpreted by the authors as consistent with—but not conclusive evidence of—the hypothesis that perception during OBEs can be externally localized.

A more sophisticated follow-up study was published in Osis and McCormick (1980), introducing a novel design to test both perceptual and possible kinetic/mechanical aspects of OBEs. The same participant was asked to leave his body and position himself in a shielded chamber containing strain-gauge sensors, placed in front of the viewing window of an optical image device that displayed visual targets. Across 197 trials, he attempted to identify the targets while the sensors recorded potential mechanical disturbances attributed to the “OB body.” Notably, strain-gauge activation was significantly greater during correct identifications than misses. Although perceptual accuracy remained modest, these kinetic findings opened a new avenue for OBE research, suggesting that future studies should explore not only information acquisition but also possible physical correlates of the projected state.

### The Palmer and Lieberman studies (1970s)

Between 1974 and 1978, Palmer (1978a) conducted a series of studies investigating ESP during OBEs and possible associated psychological or physiological traits. Using undergraduate participants, they employed randomized target pictures drawn from photography magazines and presented them under various OBE induction conditions involving relaxation, sensory deprivation, or visual and auditory stimulation. Across studies (Palmer and Lieberman, 1975a,b; Palmer and Vassar, 1974; Palmer, 1978b), no consistent evidence of ESP emerged. However, results varied with the induction procedure: when subjects experienced simple sensory deprivation, modest ESP effects appeared, whereas additional visual or tactile stimulation reversed this trend.

Notably, the authors emphasized that participants’ psychological expectations and attitudes—such as openness to ESP or OBEs—could influence their performance on ESP tasks (Palmer and Lieberman, 1975b). They found that individuals with stronger expectations of success tended to perform better, suggesting a nuanced interplay between belief, motivation, and task outcome. Although their results did not provide conclusive evidence for ESP during OBEs, they underscored the complexity of investigating such altered states and highlighted how psychological readiness and experimental context can shape outcomes.

## Limitations of traditional methodologies

In examining the historical and ongoing efforts to study the possibility of ESP during OBEs, we are increasingly confronted with the methodological limitations inherent in conventional research paradigms. Most prior studies have employed controlled protocols in sterile environments, such as requiring participants to identify alphanumeric characters or static images concealed in nearby physical locations. While these approaches provide experimental precision and ease of statistical evaluation, they inadequately reflect the phenomenological richness and attentional dynamics that experiencers consistently report.

A common limitation in current OBE research is the artificial and often unsuitable nature of the laboratory environments in which participants are asked to induce their experiences. As previously noted (Tart, 1998; Weiler et al., 2025a), participants who were otherwise experienced in self-inducing OBEs in their own natural, home conditions reported difficulty achieving or maintaining the experience in the laboratory due to external disruptions or even unfamiliarity with the environment. These findings underscore the need for researchers to carefully consider environmental factors when designing studies, as conventional laboratory settings may inadvertently suppress or alter the phenomenology of OBEs. The sterile and reductionist nature of such environments may not only fail to elicit authentic OBEs but may also suppress the depth, emotional salience, and contextual immersion often associated with veridical perception during spontaneous experiences.

A further methodological challenge concerns the mental, psychological, and emotional state of participants when attempting an OBE. Many experiencers report that their ability to disengage from the body and sustain the experience depends on entering a state of deep relaxation, trust, and openness—often preceded by meditative or ritual-like preparation. In contrast, the laboratory environment, with its time constraints, performance expectations, and potential anxiety or stress, can hinder this state and make the experience less likely to occur. Without accounting for these subtle but essential preconditions, researchers risk measuring participants’ discomfort rather than their genuine capacity for veridical perception within altered states.

The relationship between researchers and participants also plays a decisive role in shaping outcomes. Experiencers frequently report that interpersonal trust and mutual respect are essential in creating an atmosphere that supports the emergence of the experience. Conversely, if participants sense skepticism, indifference, or purely instrumental treatment, this can inhibit the altered state itself or redirect the focus of the experience away from the task. This dynamic suggests that the researcher’s role is not merely to observe and record but also to foster a relational field in which the participant feels both supported and understood. Traditional methodologies (i.e., sterile rooms, rigid protocols, and emotionally neutral researcher–participant interactions), with their emphasis on neutrality and detachment, may inadvertently undermine the very interpersonal qualities that participants identify as necessary for entering and sustaining the OBE state.

Experiencers also describe being sensitive to the researcher’s perceived intentions—whether they felt genuinely respected, supported, and understood, or scrutinized or judged. When the experiment is framed purely as a test of ESP under rigid conditions, individuals often experience the tasks as reductive or disconnected from the deeper meaning of their state. By contrast, when participants perceive that the investigators hold a genuine openness and curiosity, rather than merely a skeptical or confirmatory agenda, they report greater ease in engaging in the altered state.

The notion that the participant-experimenter relationship, as well as the experimenter’s intentions and attitudes, can influence experimental outcomes is supported by prior research in both psychology (Rosenthal, 1976) and parapsychology (Palmer and Millar, 2015). In psychological research, this influence is broadly referred to as “experimenter effects” and relates to biases arising from the expectations, behavior, or interpersonal dynamics of the experimenter, which may subtly influence participant responses and behavior and thus influence experimental results. Within parapsychology, experimenter effects have been widely recognized. Experimenters who are proponents of ESP are more likely to observe results supportive of the existence of ESP compared to skeptics, even in cases where they do not directly interact with participants in the conventional ways associated with experimenter effects (Kennedy and Taddonio, 1976; Palmer and Millar, 2015). While these effects remain controversial in parapsychology, they present an intriguing paradox (Rabeyron, 2020): the presence of this effect may weaken the strictly observational nature of experiments, but it may also provide evidence of non-local influence, if the experimenter’s own attitudes can exert an observable impact on the manifestation of the phenomenon in the laboratory. Specifically, in research on veridical OBEs, such effects may be viewed as methodological opportunities rather than limitations and can offer theoretical advancements in terms of understanding the dynamics of the OBE state.

Equally important are the skills of the participants themselves, which vary considerably and cannot be assumed to be uniform across experiencers. Just as meditators differ in depth of practice and athletes differ in training, individuals who report OBEs demonstrate varying levels of ability to induce, stabilize, and control their experiences. Yet, some previous studies have relied primarily on students or otherwise inexperienced participants, who may have little to no prior practice in inducing or sustaining OBEs (Palmer and Lieberman, 1975a,b; Palmer and Vassar, 1974; Palmer, 1978b). Expecting all participants to perform under identical conditions disregards these differences and risks flattening the data. Some may be able to direct their awareness toward specific experimental tasks, while others may only sustain brief moments of bodily disembodiment before “being swept into other dimensions of experience.” A more refined methodological approach would acknowledge these skill differentials and adapt protocols accordingly, rather than impose one-size-fits-all designs.

Another significant limitation concerns the motivation and attentional dynamics during OBEs. OBEs are events that often unfold within an altered temporal and spatial framework that differs markedly from ordinary waking consciousness (Alvarado et al., 1999). Many individuals describe the need for a finely attuned focus to maintain any connection with the physical environment; without it, their awareness tends to shift toward more abstract or “spiritual” domains. This poses a considerable challenge for experimental designs that rely on fixed, location-specific targets in the physical environment. Rather than remaining oriented toward a concealed image or object placed in the corner of a room, OBErs frequently report being drawn to experiences they perceive as “spiritually urgent” or personally significant, such as assisting others, encountering non-physical entities, or exploring alternative realities. Many participants describe OBEs not as arbitrary perceptual excursions but as meaningful, goal-oriented events—whether to gain insight, provide assistance, or explore existential questions. Within such contexts, the task of identifying a playing card on a shelf may appear not only irrelevant but profoundly misaligned with the experiential priorities of the state itself.

This limiting issue is further supported by a detailed understanding of reports from individuals who have had a near-death experience (NDE) (Charland-Verville et al., 2020; Greyson, 1983), which constitute a specific form of OBE occurring under extreme physiological conditions—such as cardiac arrest or severe trauma—when brain function is greatly impaired. NDEs are highly impactful and often spiritually transformative experiences that include intense cognitive, affective, physical, and even mystical elements (Greyson, 1983, 1985). The majority of experiencers describe in these moments a cognitive-motivational state markedly different from their typical embodied state. One key takeaway from these accounts is the individuals’ letting go of mundane tasks and obligations that feel significantly less important during their NDE, replaced with an intense desire to think about—and move toward—personally meaningful places and people, including those who are deceased (Greyson, 2021).

For those interested in studying the veridicality of OBEs, there is much to learn from NDE accounts, despite the idiosyncratic nature of these near-death OBEs. Any objective reading of these narratives quickly reveals a potential flaw in the classic, sterile laboratory approach so often employed in studies. Asking participants to maintain focus on a mundane task (i.e., find a random string of numbers) that is only meaningful to the researcher seems to be missing the point of what experiencers tell us. As researchers, we must consider that in these moments, when people perceive themselves to be disembodied, they are cognitively and emotionally motivated differently than they were before the experience. As such, attempts to test the question of veridicality require a different approach. One such suggestion (among many, no doubt) would be to include randomly occurring trials where a personally meaningful item that is brought to the lab by the participant is placed next to the target. Speculating that a participant could “truly be out-of-the-body”, we can hypothesize that they would be drawn to the target area—and thus observe the target—if they had a personal reason to go there.

With this in mind, we consider the purpose of the experience to be the most important aspect to be considered. The misalignment between the experiential purpose and the experimental task likely accounts for why so many laboratory studies have struggled to replicate the richness of spontaneous reports. Designing protocols that can integrate, or at least respect, the participant’s sense of purpose may enhance the relevance and ecological validity of the research while still permitting veridical assessment.

From a neuroscience perspective, OBEs have also been examined as disturbances of multisensory body representation. Experimental and clinical studies suggest that the sense of embodiment arises from the integration of visual, vestibular, proprioceptive, and somatosensory signals within distributed cortical networks, particularly in the temporoparietal junction and adjacent parietal regions (Lenggenhager et al., 2006). Disruptions in these integrative processes—whether through neurological lesions, electrical stimulation, sensory conflicts, or virtual-reality manipulations—can produce experiences resembling aspects of OBEs, including altered self-location, disembodiment, and changes in perspective (Blanke et al., 2002; Ehrsson, 2007; Greyson and Pehlivanova, 2025; Martial et al., 2023). Related work in cognitive neuroscience has demonstrated that experimentally induced body illusions and perspective shifts can alter perceived self-location, highlighting the malleability of bodily self-consciousness under specific perceptual conditions (Aspell et al., 2012; Maselli and Slater, 2013; Song et al., 2023). These findings have significantly advanced our understanding of the neural mechanisms underlying embodiment and altered self-experience.

At the same time, the laboratory paradigms used in these studies differ substantially from spontaneous OBEs reported in naturalistic settings. Most experimental manipulations produce brief alterations of body perception under tightly controlled sensory conditions, whereas spontaneous OBEs often occur during sleep transitions, meditation, near-death states, or other altered states of consciousness that involve complex cognitive and emotional dynamics (Moix et al., 2025; Weiler et al., 2024). As a result, while neuroscience research provides valuable insights into mechanisms of body representation and perceptual integration, it does not fully address the broader phenomenological and contextual features reported in many spontaneous OBEs. Recognizing these differences underscores the importance of developing research approaches that integrate insights from neuroscience with methodologies capable of capturing the experiential dynamics of these states.

Taken together, these considerations point to the limitations of applying traditional laboratory-based methodologies to OBE research without adapting them to the psychological, interpersonal, and phenomenological realities of the state. By privileging experimental control over experiential authenticity, we risk overlooking the very conditions under which possible ESP might most plausibly occur. Future designs will need to integrate these elements, creating environments and protocols that are both scientifically rigorous and experientially attuned if we are to meaningfully assess claims of ESP during OBEs. To reduce the risk of interpretive bias when examining spontaneous reports, future research should prioritize structured documentation protocols, including time-stamped recording of experiences, independent verification of reported details, and systematic evaluation of possible ordinary sensory pathways.

## Advancing the field: leveraging anecdotal reports as evidence of ESP

A promising approach for investigating the possibility of ESP during OBEs is to examine anecdotal reports arising from spontaneous experiences. Although anecdotal by nature, certain characteristics of these reports can offer meaningful support for hypotheses related to ESP. When evaluated against appropriate evidentiary standards, such cases may serve as valuable data points for developing empirically grounded models of non-local consciousness. Examples include detailed descriptions of spatial features in locations the experiencer had never physically visited, as well as accurate accounts of events unfolding in real time that were subsequently confirmed by independent sources. These phenomena are especially compelling when the reported information was demonstrably unknown to the experiencer at the time of the OBE. In certain instances, individuals even report accurately perceiving events, environments, or interpersonal interactions that took place while they were unresponsive, asleep, or otherwise physically incapacitated.

Additionally, in some instances, multiple individuals report congruent experiences during OBEs, including shared perceptions or mutually corroborated interactions. These accounts may involve one individual reporting an OBE in which they intentionally visited another person, who independently reports sensing or perceiving the presence of the experiencer. In other cases, two or more individuals describe overlapping or identical features of a non-physical environment encountered during their respective altered states. When such reports are supported by independent testimony and temporal alignment, they suggest the possibility of ESP elements within the OBE, warranting further systematic investigation.

In other reports, individuals who are blind, anesthetized, or otherwise deprived of normal sensory input nonetheless describe accurate details of their surroundings or external events during an OBE. These accounts often include vivid and specific observations that, under ordinary conditions, would require intact sensory functioning. Documented examples include surgical patients under general anesthesia who later accurately describe the appearance of medical personnel, the layout of the operating room, or the sequence of procedures performed (Mitchell, 1973; van Lommel et al., 2001). [Although research on “connected consciousness” demonstrates that covert awareness can persist during behavioral unresponsiveness—including under general anesthesia, where patients may still process verbal information or form memories (Sanders et al., 2012; Wang et al., 2021)—this phenomenon does not account for the spatially displaced, vantage-point–dependent perceptions reported in many OBEs and NDEs. Rather, it underscores that subjective awareness can be preserved even under conditions of profound physiological suppression, while leaving unexplained how some individuals acquire accurate information that exceeds what is accessible through covert sensory processing alone].

Similarly, some congenitally or temporarily blind individuals have reported detailed visual perceptions during OBEs that correspond closely to real-world stimuli (Davis-Cambridge, 1976; Irwin, 1987; Ring and Cooper, 1997). Such cases raise important questions regarding the necessity of functional sensory pathways for accurate perception and challenge conventional assumptions about the sensory basis of consciousness, as well as pointing to the possibility of non-local mechanisms of information access.

## Criteria for evaluating ESP claims in spontaneous OBEs

In legal contexts, eyewitness testimony is routinely accepted as legitimate evidence, despite being anecdotal, subjective, and retrospective. Courts acknowledge that firsthand accounts—though not produced under controlled conditions—can provide critical information about events that cannot be directly replicated. This legal precedent highlights the value society places on detailed personal narratives when objective verification is limited. Applying this logic to the study of spontaneous OBEs, anecdotal reports containing verifiable information should not be dismissed outright but instead assessed using appropriate evidentiary standards. As with crime scene investigations, evaluating the credibility of such reports requires a thorough and methodical approach.

To minimize interpretive bias and increase methodological transparency, the evaluation of spontaneous OBE reports should rely on clearly defined criteria applied systematically across cases. The following considerations provide a structured framework for distinguishing reports that may contain evidential elements from those more plausibly explained by conventional mechanisms.

### Accurate temporal documentation

Accurate temporal documentation of when the experience occurred—and when the information was reported—is critical. Ideally, the content of the OBE should be recorded before any opportunity for post hoc validation (e.g., through conversations, media, or subsequent exposure). Time-stamped written or audio records can provide essential safeguards against contamination by later-acquired knowledge. The clearer the temporal sequence between the OBE, the report, and the confirmation of facts, the stronger the evidentiary value. The use of the current time as a visual experimental target displayed in a concealed location can be useful in establishing time anchors (Greyson et al., 2006).

### Independent verification

Credibility is significantly enhanced when the details reported by the experiencer can be confirmed by independent sources who had no prior involvement in or influence over the OBE. Verification should ideally be conducted by neutral third parties—such as medical personnel, investigators, or unrelated witnesses—who can attest to the accuracy of specific observations or events described. This process should be as objective and documentation-driven as possible, minimizing interpretive bias or retrospective reconstruction. Independent verification is also important to control for psychological artifacts, such as confabulation, expectation effects, suggestibility, and memory distortion, that can give rise to seemingly paranormal reports.

### Exclusion of ordinary sensory pathways

For claims of ESP to be considered plausible, it must be demonstrated that the reported information could not reasonably have been obtained through ordinary sensory channels. Researchers should investigate factors such as line-of-sight visibility, auditory leakage, prior knowledge, or indirect suggestion. In cases involving medical scenarios, for example, patient access to procedural information via ambient conversation or prior experience must be carefully ruled out. Similar considerations have been formalized in a recently published veridical NDE scale (Greyson et al., 2025), which provides a systematic and standardized tool for assessing the evidential strength of perceptions during this state.

Taken together, these criteria allow spontaneous OBE cases to be evaluated using a form of methodological triangulation. When applied systematically, these criteria enable researchers to identify cases of spontaneous OBEs that merit further empirical investigation. If anecdotal testimony is deemed sufficiently credible to serve as legal evidence—sometimes even determining the outcome of criminal trials—it stands to reason that well-documented and rigorously evaluated accounts of veridical OBEs should also be granted serious consideration, even in the absence of laboratory replication.

## Conclusion

The veridicality of OBEs remains a fascinating question, hindered largely by methodological limitations. While traditional paradigms have provided valuable insights, they may be insufficient to test the full range of experiences reported by OBErs. By adopting emotionally grounded dyadic studies and alternative methodologies, such as well-investigated spontaneous cases, researchers may gain a deeper understanding of the perceptual mechanisms and epistemological implications of OBEs.

## Data Availability

The original contributions presented in this study are included in this article/supplementary material, further inquiries can be directed to the corresponding author.
